# A novel bidirectional LSTM deep learning approach for COVID-19 forecasting

**DOI:** 10.1038/s41598-023-44924-8

**Published:** 2023-10-20

**Authors:** Nway Nway Aung, Junxiong Pang, Matthew Chin Heng Chua, Hui Xing Tan

**Affiliations:** 1 Institute of Systems Science, National University of Singapore, 25 Heng Mui Keng Terrace, Singapore, 119615 Singapore; 2 Saw Swee Hock School of Public Health, National University of Singapore, Singapore, Singapore; 3 Department of Biomedical Informatics, Yong Loo Lin School of Medicine, National University of Singapore, 1E Kent Ridge Road, Singapore, 119228 Singapore; 4 Centre for Outbreak Preparedness, SingHealth Duke-NUS Global Health Institute, Duke-NUS Medical School, NUS, Singapore, Singapore

**Keywords:** Infectious diseases, Scientific data

## Abstract

COVID-19 has resulted in significant morbidity and mortality globally. We develop a model that uses data from thirty days before a fixed time point to forecast the daily number of new COVID-19 cases fourteen days later in the early stages of the pandemic. Various time-dependent factors including the number of daily confirmed cases, reproduction number, policy measures, mobility and flight numbers were collected. A deep-learning model using Bidirectional Long-Short Term Memory (Bi-LSTM) architecture was trained on data from 22nd Jan 2020 to 8 Jan 2021 to forecast the new daily number of COVID-19 cases 14 days in advance across 190 countries, from 9 to 31 Jan 2021. A second model with fewer variables but similar architecture was developed. Results were summarised by mean absolute error (MAE), root mean squared error (RMSE), mean absolute percentage error (MAPE), and total absolute percentage error and compared against results from a classical ARIMA model. Median MAE was 157 daily cases (IQR: 26–666) under the first model, and 150 (IQR: 26–716) under the second. Countries with more accurate forecasts had more daily cases and experienced more waves of COVID-19 infections. Among countries with over 10,000 cases over the prediction period, median total absolute percentage error was 33% (IQR: 18–59%) and 34% (IQR: 16–66%) for the first and second models respectively. Both models had comparable median total absolute percentage errors but lower maximum total absolute percentage errors as compared to the classical ARIMA model. A deep-learning approach using Bi-LSTM architecture and open-source data was validated on 190 countries to forecast the daily number of cases in the early stages of the COVID-19 outbreak. Fewer variables could potentially be used without impacting prediction accuracy.

## Introduction

Coronavirus disease 2019 (COVID-19) is a global public health crisis declared a pandemic by the World Health Organization. As of March 2021, the virus had infected over 127.6 million people worldwide and the number of deaths had totaled more than 2.7 million ^1^. Compared to other highly contagious previously identified coronavirus-related diseases, such as Severe Acute Respiratory Syndrome (SARS) and Middle East Respiratory Syndrome (MERS), the SARS-CoV-2 virus that resulted in COVID-19 disease appears to be more infectious. It is critical to explore novel approaches to monitor and forecast regional outbreaks in the early phase of the pandemic in order to facilitate better allocation of resources and containment planning ^1–3^ by healthcare providers and policymakers.

A crucial part of planning in this scenario is forecasting the daily confirmed cases of COVID-19. In the short-term, predictions can be performed by time series analysis ^2^. With the rapid spread of COVID-19, various forecasting, estimation, and modelling approaches are introduced. For instance, to forecast the evolution of confirmed infected cases, both epidemiological models of SIR and SER were used. In the early stages of the epidemic, a single individual can infect several people before isolation but raising public awareness, health, and stringency control, as well as policy controls and movement restrictions, may help control the epidemic. Reproduction Number (Rt), which is characterized by the number of people caused by a single individual at each stage of the outbreak, can also determine the different stages of the infection outbreak. The effective reproduction number of SIR model is used to assess the progress of the epidemic^3^. Using the forecast for the number of infected (I), recovered (R), and dead (D) individuals, Rt and its temporal evolution are computed. The SIR computes the theoretical number of individuals infected with a contagious illness in a closed population over time with three states: Susceptible people S(t), Infected I(t), and Recovered R(t)^4^. The susceptible exposed infectious recovered model (SEIR) models population are classified into four categories: S (Susceptible), E (Exposed), I (Infected), and R (Recovered) according to the states of individuals ^5,6^. In ^7^, the SIR model outperforms the SEIR model in terms of Akaike Information Criteria (AIC) to forecast and predict the confirmed cases data information.

Some preliminary studies for COVID-19 time series forecasting using Autoregressive Integrated Moving Average (ARIMA) methods have also been done ^8–10^. Many types of research based on traditional time series forecasting models have been explored to forecast future COVID cases ^11–13^. Machine learning and deep learning have developed as promising research ^14–16^ in accurately predicting the number of confirmed COVID-19 cases. In China, a stacked auto-encoder model is designed to fit the epidemic's dynamical propagation and real-time forecasting of confirmed cases ^17^. For forecasting using time series analysis, deep-learning using recurrent neural networks, or RNN, are proposed as promising methods to predict the risk category trend predictions ^18,19^. Overall, there have been many developments in the prediction of COVID-19 cases, including the use of LSTM approaches ^14,18,20,21^ However the analyses are limited to a number of countries (China, India, US, Canada, Australia, and European Countries) and no data on external factors such as containment measures are used in the forecasts. Furthermore, most of the studies use mean squared error (MSE) or mean absolute error (MAE) as a way to evaluate the performance of the models in a single country, which may not be applicable when comparing model performance across multiple countries.

## Objectives

**Figure 1:**
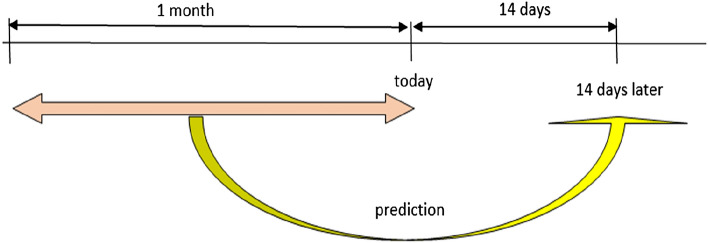
Use of 30 days prior data to predict the number of new cases 14 days later.

The US CDC has adopted an ensemble forecasting method ^22^ to generate 4-week forecasts for the number of deaths and confirmed cases and evaluated that accuracy of the model deteriorated at longer prediction horizons of up to four weeks. According to the US CDC ^23^, the number of deaths and confirmed cases is seen to fall within 30 days after containment measures are taken. We aim to use data from thirty days before a fixed time point to forecast the number of daily cases fourteen days later, which would be a reasonable time frame to facilitate planning dictions (Fig. 1). As various time-dependent factors including the number of daily confirmed cases, reproduction number, containment, and governmental policy measures, mobility and flight data could affect the daily number of cases in the future, these data, where available, were included in the analyses.

## Methods

### Datasets

Data on daily new cases from the earliest date to 22 Jan 2020 to 31 Jan 2021 were collected from the Johns Hopkins University research databases (https://github.com/CSSEGISandData/COVID-19) ^24^. Numerical data on twenty-four time-dependent variables of 190 countries were collected from various sources such as the website ourworldindata.com ^25^. Flight data, where available, were collected from the Official Airline Guide (OAG) ^26^. Effective Rt is a well-known parameter to evaluate the propagation of the outbreak and is thus used as one of the input variables to predict the daily confirmed cases in this study. The computation of effective Rt is adopted from ^3^. The details of these variables are listed in Table 1.

**Table 1:** Variables collected for timeseries analysis and their sources.

Description	Frequency of refresh	Data source
Infected covid cases	Daily	COVID tracking data from Johns Hopkins Coronavirus Resource Center
Dead covid cases		
Recovered covid cases		
Derived data on the effective reproduction number, Rt	Daily	"Country-wise forecast model for the effective reproduction number Rt of coronavirus disease," *Frontiers in Physics,* vol. 8, p. 304, 2020
Flight data for 12 countries -United states -United Kingdom -UAE -Germany -Spain -France -Japan -Korea South -China -Brazil -Sweden -Singapore	Weekly	Flight Data from Official Airline guide (OAG) website
Covid test data, total tests, and per thousand population	Daily	Testing data from ourworldindata.com
Level of containment policies (international travel controls, contact tracing, facial coverings, stay-home requirements) adopted by each country across the time	Daily	Containment policies from ourworldindata.com
Mobility data - This new dataset from Google measures visitor numbers to specific categories of location (e.g. grocery stores; parks; train stations) every day and compares this change relative to the baseline day before the pandemic outbreak. Baseline days represent a normal value for that day of the week, given as the median value over the five-week period from January 3rd to February 6th, 2020. Measuring it relative to a normal value for that day of the week is helpful because people obviously often have different routines on weekends versus weekdays	Daily	Google mobility data from ourworldindata.com

The dataset is updated daily with new information. For this experiment, all data from 22 January 2020 to 31 January 2021 were used. Time series points with a missing numbers were replaced with 0.

### Overview of approach

**Figure 2:**
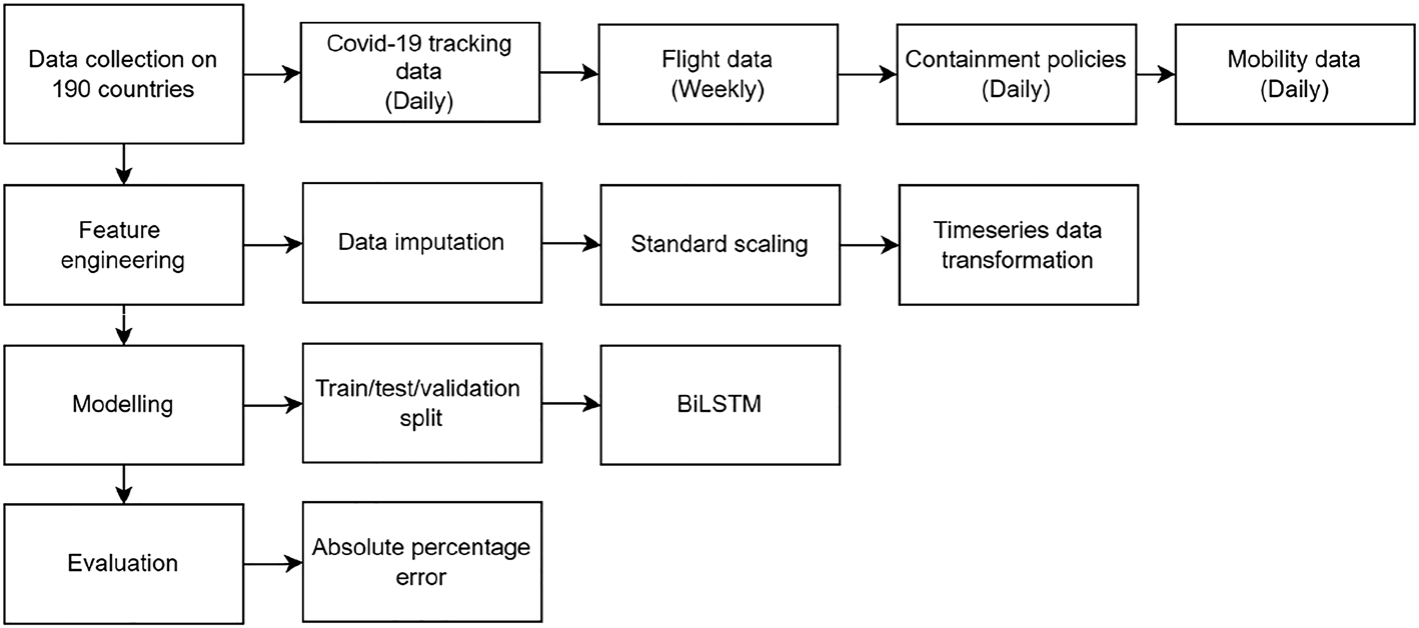
Conceptual framework of the proposed forecasting methods.

This study proposes a deep-learning framework for COVID-19 time-series prediction. The framework is illustrated in Fig. 2.

### Feature engineering

Data pre-processing is one of the crucial steps in machine learning. The time-series data of 190 countries were collected, pre-processed, and analysed for each country. Standard scaling was applied using StandardScaler() in Python 3.7 to scale each of the 24 variables to zero mean and unit variance. As the model requires a sequence of past observations as input and maps it to the output observation, thirty days of time steps up till the current day are used as input, and a one-time step of the target variable fourteen days later is used as output for the one-step prediction that is being learned.

### Modelling

Modelling is done individually for each country and has been done in two main stages: the training and testing stage. Data for the training stage comprised data from 22 January 2020 to 8 Jan 2021 for a total of 353 days, and data for the testing stage span the period from 9 Jan 2021 to 31 Jan 2021. The raw data is pre-processed, standardized, and then used to build the deep learning model.

### BiLSTM

**Figure 3:**
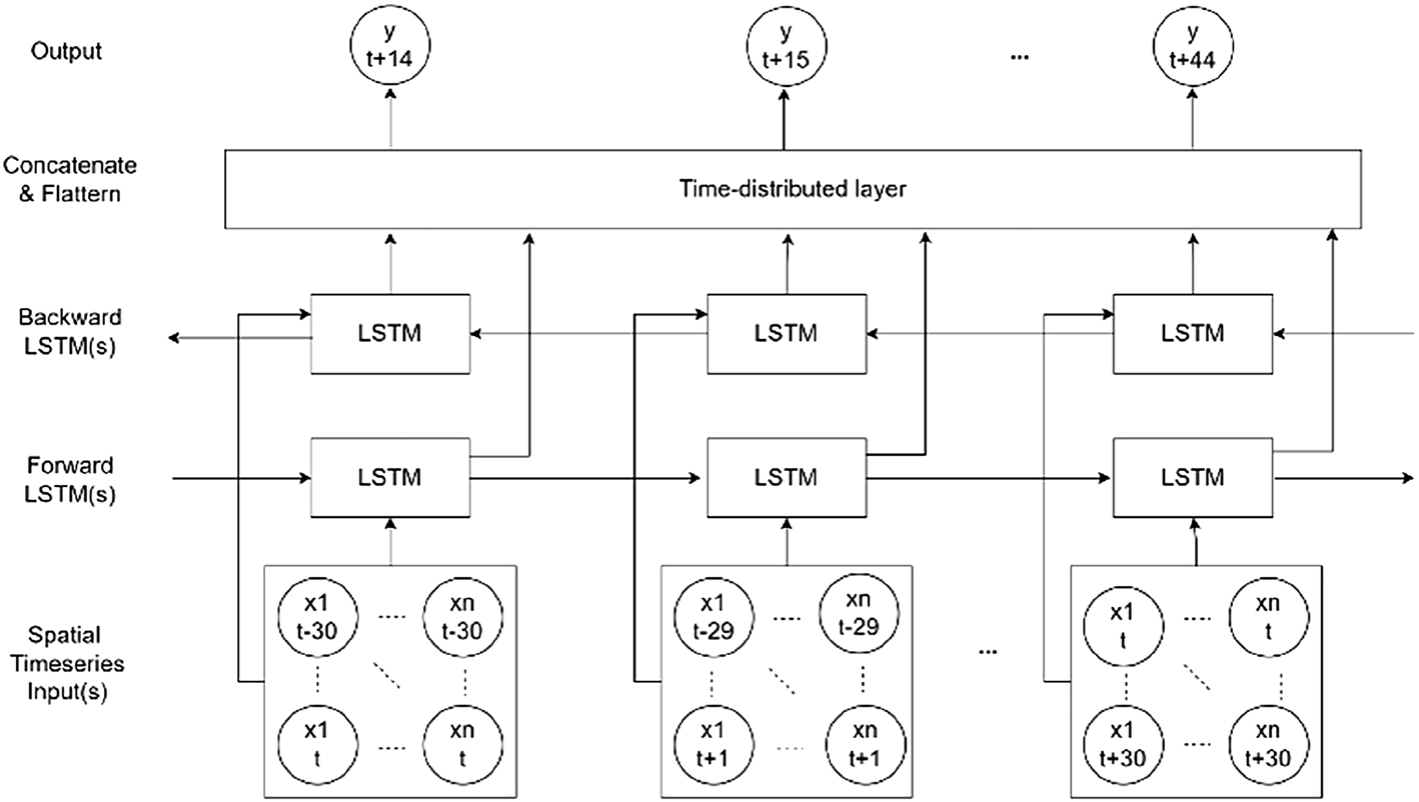
Modelling with Bidirectional long short-term memory networks.

Time series of daily new confirmed COVID-19 cases were used for generating 14-day forecasts using Bidirectional Long-short Term Memory models (BiLSTM). A BiLSTM is an enhanced version of the LSTM algorithm. LSTMs were designed to process sequences of data and improved upon traditional RNN by using memory cells that can store information in memory for long series and a set of gates to control the flow of this memory information. These innovations allow LSTM to learn longer-term dependencies in sequential data. One of the limitations of LSTM is that the current state can only be reconstructed through the backward context. The BiLSTM algorithm fuses the ideal functions of bidirectional RNN and LSTM. This is done by combining two hidden states, which allow information to come from the backward layer and the forward layer. The BiLSTMs were trained on varying sizes of input sequences—sequence sizes of 128, 64, and 20. Detailed modelling can be seen in Fig. 3.

### Hyper-parameter tuning

Hyper-parameter tuning is conducted with trial and error during the training. In the experiments, rmsprop optimizer with a learning rate of 0.1 was used for training the LSTM models, and the mean absolute error was used as the loss function. After that, the models with the selected hyper-parameters were used to forecast the number of COVID cases in the testing stage. The model's accuracy was verified by comparing the measured data with real data via different statistical indicators, including Root Mean Square Error (RMSE), Mean Absolute Error (MAE), Mean Absolute Percentage Error (MAPE) and total absolute percentage error (see evaluation metric).

### Features used

**Figure 4:**
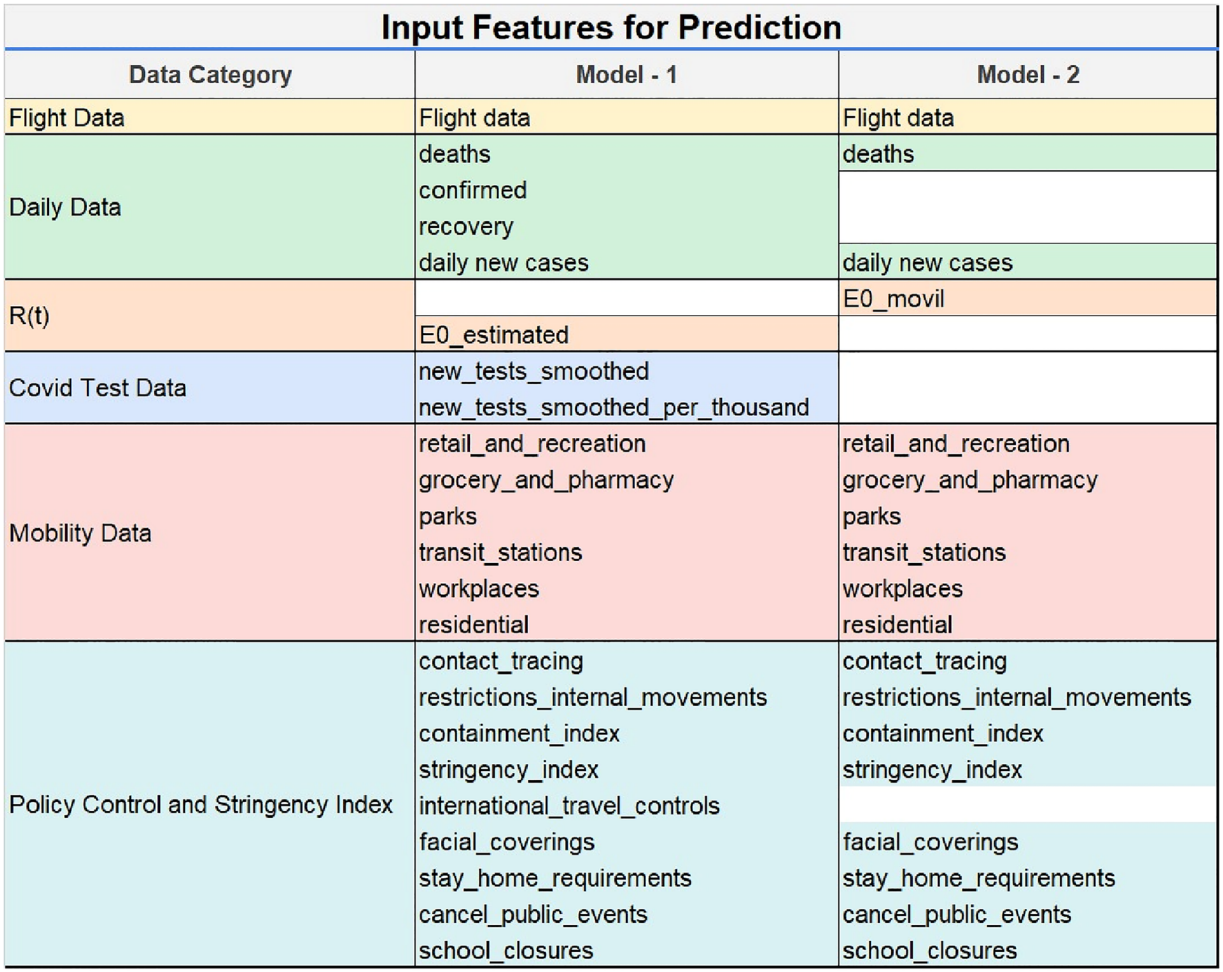
Input features used for prediction. Key: flights—daily number of flights; deaths—cumulative number of COVID-19 deaths, confirmed—cumulative number of confirmed cases; recovery—cumulative number of recovered cases; E0_movil—daily reproduction number, Rt, smoothed; E0_estimated—daily reproduction number, Rt; new_tests_smoothed—daily test numbers; new_tests_smoothed_per_thousand—daily test numbers per thousand population; retail_and_recreation, grocery_and_pharmacy, parks, transit_stations. workplaces, residential—mobility data from Google contact_tracing—level of contact tracing (3 levels); restrictions_internal_movements—restrictions on internal movement during the COVID-19 pandemic (3 levels); containment_index—Containment and Health Index, a composite measure of eleven response metrics; stringency index—Government Stringency Index, a composite measure of nine response metrics; international_travel_controls—government policies on restrictions on international travel controls. (5 levels); facial_coverings—use of face coverings outside-of-the-home; stay_home_requirements—government policies on stay-at-home requirements or household lockdowns; cancel_public_events—government policies on the cancellation of public events; school_closures—government policies on school closures.

Two sets of input features were used for each model as shown in Fig. 4. The first model (Model 1) used all features except for the computed Rt moving average. The second model (Model 2) used all features except the confirmed and recovered cases (as the information was partially captured in daily new cases), estimated Rt (as the information was partially captured in Rt moving average), number of new tests done each day, as well as international travel controls (as the information was partially captured in flight data).

### Evaluation metric

In addition to RMSE, MAE and MAPE, total absolute percentage error was used for evaluating the performance of the models as shown.

$${\text{Total absolute percentage error}} = \, \left| {{\text{TotalActual }}{-}{\text{ TotalPredicted}}} \right| \, \div {\text{TotalActual}} \times { 1}00\%$$

where TotalActual and TotalPredicted refer to the actual and predicted sum of total new cases over the testing period, respectively. A sub-analysis was performed in 84 countries with more than 10,000 cases over the predicted period (or an average of 434 new cases a day) as percentage error could be inflated for models with very few cases and projections would be more useful for capacity planning for countries with a large number of cases. The top five countries with the best and worst performance were analysed in terms of the number of cases per day, phase of infection and number of infections waves experienced.

### Comparison with classical ARIMA model

To test the effectiveness of the new method, an Autoregressive Integrated Moving Average (ARIMA) model was used to generate predictions over the same period using only daily new cases as inputs. Results from the classical model were summarised using the same evaluation metrics as the other two models.

### Ethics approval

This study is supported by NUS-IRB-2020-812 under National University of Singapore.

## Results

### Summary by MAE, RMSE, MAPE, percentage error

Median MAE was 157 new daily cases (IQR: 26–666) under the first model, 150 (IQR: 26–716) under the second model, and 130 (IQR: 22–475) under the ARIMA model (Table 2). The countries and their respective performance with Models 1 and 2 are listed in Supplementary Material 1. However, the effectiveness of the model is hard to gauge with MAE and RMSE alone as some countries may report thousands of daily infections, while some others only a handful of cases a day. As seen in Table 3, the worst-performing countries are countries with large numbers reported. For over half of the countries, the percentage error in terms of the total number of cases over the predicted period was at most 51% for the first model, 53% for the second model, and 41% for the ARIMA model However, the maximum error was higher under the ARIMA model as compared to models 1 and 2.

**Table 2:** Summary by MAE, MSE, percentage error.

	Model 1 (more variables)				Model 2 (fewer variables)			Classical model (ARIMA)		
	MAPE	MAE	RMSE	Percentage error (%)	MAE	RMSE	Percentage error (%)	MAE	RMSE	Percentage error (%)
Min	0	0	0	0	0	0	0	0	0	0
25P	0	26	35	22	26	37	24	22	32	15
Median	1	157	209	51	150	192	53	130	161	41
75P	4.47E+15	666	752	87	716	863	92	475	532	69
Max	1.81E+19	44,250	52,940	16,928	47,506	55,796	16,633	88,363	98,098	1296

**Table 3:** Top 5 countries with best and worst performance for each model.

		MSE	MAE	Percentage error
Best performance	Model 1	1. Vanuatu 2. Marshall Islands 3. Solomon Islands 4. Western Sahara 5. MS Zaandam	1. Vanuatu 2. Marshall Islands 3. Solomon Islands 4. Western Sahara 5. Micronesia	1. Singapore 2. Montenegro 3. Belgium 4. Congo Kinshasa 5. Slovakia
Best performance	Model 2	1. Solomon Islands 2. Vanuatu 3. Marshall Islands 4. Western Sahara 5. MS Zaandam	1. Vanuatu 2. Marshall Islands 3. Western Sahara 4. Solomon Islands 5. Micronesia	1. Pakistan 2. Germany 3. Bhutan 4. Estonia 5. Slovakia
Worst performance	Model 1	1. United States 2. Spain 3. United Kingdom 4. Brazil 5. France	1. United States 2. United Kingdom 3. Spain 4. Brazil 5. Turkey	1. Tajikistan 2. Central African Republic 3. Uganda 4. Cyprus 5. Australia
Worst performance	Model 2	1. United States 2. Spain 3. Brazil 4. United Kingdom 5. France	1. United States 2. Spain 3. Brazil 4. United Kingdom 5. France	1. Tajikistan 2. Azerbaijan 3. Trinidad and Tobago 4. Switzerland 5. Iceland

Analysis of the countries with the best and worst results in terms of percentage error shows that countries with better performing results typically had more cases per day and had witnessed more waves of COVID outbreaks over the training period (Table 4). Countries that fared more poorly had fewer daily cases and were usually not in the middle of any covid wave. This is because smaller daily cases would lead to larger percentage errors due to low base effects.

**Table 4:** Top 5 countries with the best and worst performance by percentage error with Models 1 and 2.

Model 1					
Top 5 countries with best results		Average cases per day during prediction period	In or out of infection wave	Part of infection wave	Number of infection waves
1	Singapore	30	Out	NA	2
2	Montenegro	438	In	Increasing	4
3	Belgium	2150	Out	NA	2
4	Congo Kinhasa	165	In	Declining	2
5	Slovakia	2120	In	Declining	2

Top 5 countries with worst results		Average cases per day during prediction period	In or out of infection wave	Part of infection wave	Number of Infection waves
1	Tajikistan	0	Out	NA	1
2	Central African Republic	1	Out	NA	1
3	Uganda	99	In	Declining	2
4	Cyprus	183	In	Declining	2
5	Australia	10	Out	NA	2

Model 2					
Top 5 countries with best results		Average cases per day during prediction period	In or out of infection wave	Part of infection wave	Number of infection waves
1	Pakistan	2,040	In	Declining	2
2	Germany	13,914	In	Declining	2
3	Slovakia	2120	In	Declining	2
4	Estonia	507	In	Declining	2
5	Bhutan	4	In	Declining	3

Top 5 countries with worst results		Average cases per day during prediction period	In or out of infection wave	Part of infection wave	Number of Infection waves
1	Tajikistan	0	Out	NA	1
2	Azerbaijan	296	In	Declining	2
3	Trinidad and Tobago	15	Out	NA	2
4	Switzerland	1884	In	Declining	2
5	Iceland	6	Out	NA	3

### Sub-analysis in countries with more than 10,000 cases

A sub-analysis was performed in 84 countries with more than 10,000 cases over the predicted period (or an average of 434 new cases a day). The median percentage error in terms of the total number of cases over the predicted period was lower when limited to these countries—at 33% for the first model with more variables, and 34% for the second model with fewer variables (Table 5). The percentage error was 16% or less in a quarter of cases in the first model, while the maximum error was 166% for the first model and 191% for the second. While the median percentage error was similar in the ARIMA model (32%, IQR 11–53%), Model 1 (33%, IQR 18–59%), and Model 2 (34%, IQR 16–66%), the maximum error was greater under the classical ARIMA model (462%) than under the other two models (166%, 192%).

**Table 5:** Summary by MAE, MSE, Percentage Error (countries with more than 10,000 total cases over predicted period).

	Model 1 (more variables)				Model 2 (fewer variables)			Classical Model (ARIMA)		
	MAPE	MAE	RMSE	Percentage error (%)	MAE	RMSE	Percentage error (%)	MAE	RMSE	Percentage error (%)
Min	0	56	68	0	66	78	0	50	65	0
25P	0	422	486	18	403	478	16	290	363	11
Median	1	710	881	33	865	969	34	581	696	32
75P	1	2085	2580	59	2412	2786	66	2625	3423	53
Max	1.81E+19	44,250	52,940	166	47,506	55,796	191	88,363	98,098	462

### Top 5 countries with best and worst model performance

Table 6 summarises the characteristics of the top 5 countries with the best and worst model performance. After limiting the analysis to countries with more than 10,000 cases over the prediction period, both the sets of top-performing and worst-performing models comprised countries from various regions, and most countries in the declining phase of an infection wave. However, the countries which fared better under models 1 and 2 had experienced a greater number of infection waves by the time of prediction and still had a slightly greater number of cases per day. In addition, model 2 appeared to have better performance in countries with a greater number of daily cases per day. As model 2 excluded the use of variables such as the number of tests done, it might appear that such variables are less useful and could introduce noise when the number of cases was relatively high. Model 1 appeared to produce better predictions over a greater variety of trends in daily cases (increasing, out of COVID wave, declining cases), which could suggest better generalizability across country profiles.

**Table 6:** Top 5 countries (more than 10,000 total cases over prediction period) with best and worst performance by percentage error with Models 1 and 2.

Model 1					
Top 5 countries with best results		Average cases per day during prediction period	In or out of infection wave	Part of infection wave	Number of infection waves
1	Montenegro	438	In	Increasing	4
2	Belgium	2150	Out	NA	3
3	Slovakia	2120	In	Declining	2
4	Paraguay	836	In	Increasing	2
5	Estonia	507	In	Declining	2

Top 5 countries with worst results		Average cases per day during prediction period	In or out of infection wave	Part of infection wave	Number of Infection waves
1	Turkey	7386	Out	NA	1
2	Denmark	869	In	Declining	2
3	Malawi	697	In	Increasing-peak-declining	1
4	Ghana	502	In	Increasing	2
5	Mozambique	790	In	Increasing	2

Model 2					
Top 5 countries with best results		Average cases per day during prediction period	In or out of infection wave	Part of infection wave	Number of infection waves
1	Pakistan	2040	In	Declining	2
2	Germany	13,914	In	Declining	2
3	Estonia	507	In	Declining	2
4	Slovakia	2120	In	Declining	2
5	United Kingdom	37,476	In	Declining	3

Top 5 countries with worst results		Average cases per day during prediction period	In or out of infection wave	Part of infection wave	Number of infection waves
1	Switzerland	1884	In	Declining	2
2	Croatia	630	In	Declining	1
3	Denmark	869	In	Declining	2
4	Georgia	960	In	Declining	1
5	Jordan	943	In	Declining	1

**Figure 5:**
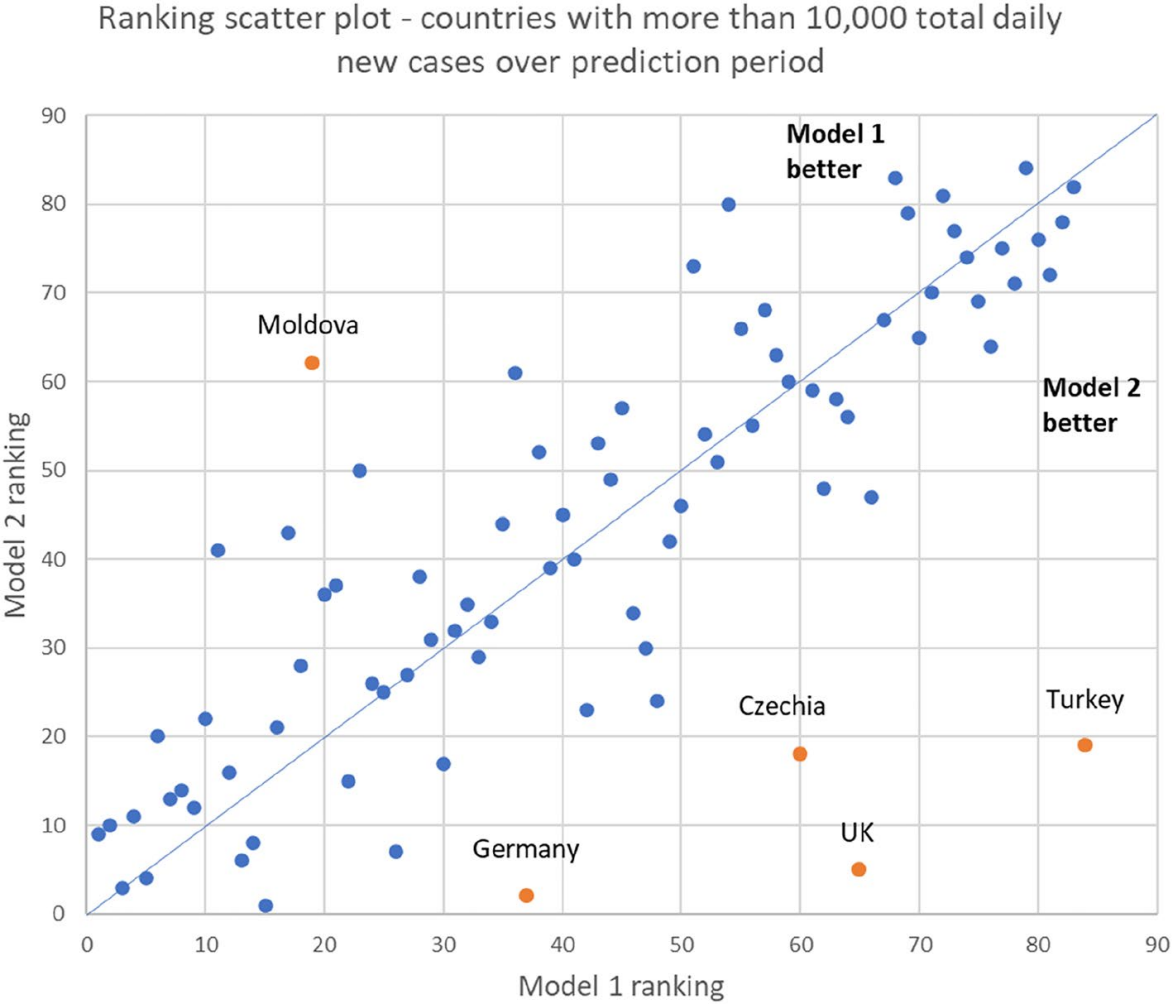
Ranking scatterplot of 84 countries.

**Figure 6:**
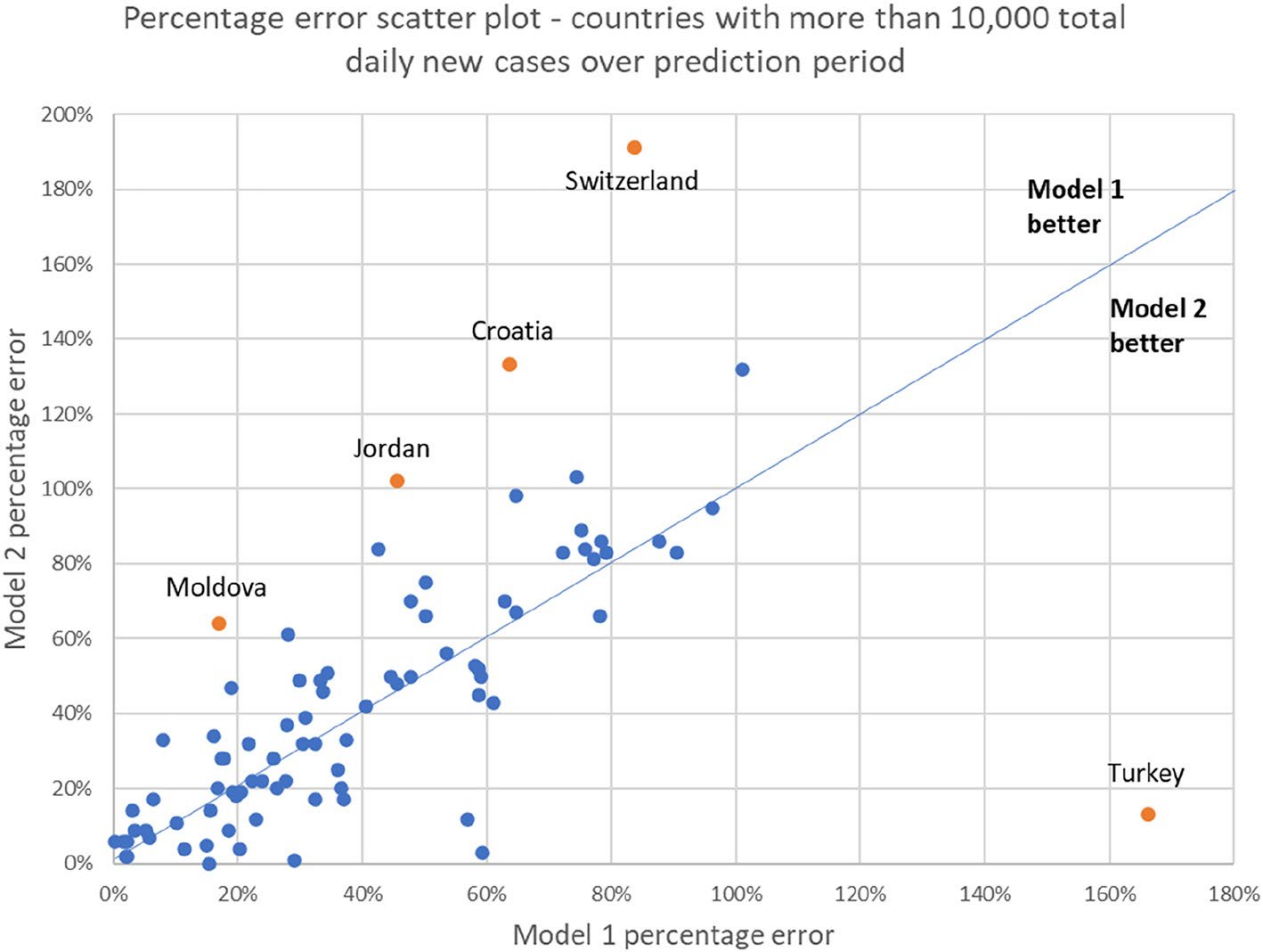
Percentage error scatterplot of 84 countries.

Figures 5 and 6 show the scatter plot of ranking in terms of percentage error, and absolute percentage error respectively. There were some countries in which Model 1 performed better (Moldova, Jordan, Croatia, Switzerland), and which Model 2 performed better (Czechia, UK, Turkey, Germany). The charts showing the predictions and actual cases for these seven countries are in Supplementary Material 2. In both cases, the poorer-performing model tended to overpredict the number of cases. However, Models 1 and 2 performed more poorly under different scenarios. Model 1 wrongly predicted that the rise in cases would continue when cases were at the peak and about to decline (Czechia, UK), while Model 2 provided higher estimates during a declining phase of infections (Moldovia, Jordan, Croatia, Switzerland).

## Limitations and strengths

The models' limitations are their restricted applicability only on some outbreak stages and with the availability of enough data. During model development, it was assumed that the intensity and coverage of surveillance and testing were consistent throughout the whole period as well as across the different countries, which realistically may not be possible due to a potential shortage of resources. With the roll-out of vaccination programmes, daily number of new cases is expected to decrease with the same input variables. Therefore, the validation was only performed using data collected before 31 January 2021, when the vaccination campaign has just started globally. On the other hand, the emergence of new virus variants with different transmissibility could also impact the performance of the models. The Omicron variant had become the dominant strain of the virus and is known to be more transmissible but less deadly. Nevertheless, the availability of open-source data and previous training of the model developed may make it useful in forecasting for outbreaks of a similar nature, especially during the early stages of an outbreak. The model with fewer inputs performed reasonably well compared to the model with more inputs, suggesting that in the case of fewer data available, a reasonable forecast could still be obtained. Nevertheless, given the varying results, it is recommended that individual models with individual sets of variables be trained specifically in those countries, using all variables as a starting point.

The strength of the models is that they draw upon readily available data on a country’s national, healthcare, social and economic status to generate predictions, and have been validated on 84 countries with more than 10,000 total cases over the prediction period that are different geographically, politically, and culturally. By running the model on 84 countries, an estimate of the maximum possible error is obtained, allowing for planning of best and worst case scenarios. An additional strength of the models is the ability to generate predictions fourteen days in advance, without knowledge of the number of cases or changes in the upcoming thirteen days. As such, these predictions would be useful for facilitating the better allocation of resources and containment planning by healthcare providers and policymakers over a longer time horizon.

## Discussion

Previous work has been done on COVID-19 forecasting using both classical and machine learning methods. Miralles-Pechuán et al. compared the performance of state-of-the-art machine learning algorithms, such as long-short-term memory networks, against that of online incremental machine learning algorithms to predict the coronavirus cases for the 50 countries with the most cases during 2020 ^27^. Kasilingam et al. used exponential growth modelling studies to understand the spreading patterns of SARS-CoV-2 and identify countries that showed early signs of containment until March 26, 2020 ^28^. Saba et al. applied time-series and machine learning models to forecast daily confirmed infected cases and deaths due to COVID-19 for countries under various types of lockdown (partial, herd, complete) ^29^. While these studies used machine learning and acknowledged the impact of containment measures on the daily case numbers, our study is novel in terms of the variates used and approach.

To our best knowledge, this study is the first that leverages open-source data including flight data to perform COVID-19 time series forecasting on 190 countries using a machine learning approach. Only data up to 31 January 2021 could be accessed at the time the analysis was conducted. The model was able to predict the total number of cases across a period of 14 days in advance from 9 Jan 2021 to 31 Jan 2021 with a median of 35% error amongst countries with more than 10,000 cases over the predicted period, or an average of 434 new cases per day. When tested on countries with more than 10,000 cases over the predicted period, maximum error was much smaller for the Bi-LSTM model than a classical ARIMA model, suggesting that using more variables and machine learning methods could help to minimise the maximum error.

The model was developed in 190 countries but validated over 84 countries with more daily cases. Further fine-tuning of the models to create a country-specific model is warranted given the varying results across different countries.

Upon analysis of the key characteristics of the top five countries with best and worst performance found that countries with the best performance in terms of percentage error had experienced more waves of COVID-19 infections prior, such that the prediction method would be more suitable for countries who had more historical data for training on. Cases were on the decline for most countries, and thus the model might be better in prediction when the trend in daily cases is stable and less accurate in predicting sudden surges 14 days in advance.

The models in our study were trained and tested in isolation on each country, that is, model weights obtained from training on one country were not used for prediction on another country. Given that the models performed relatively well on countries which had experienced earlier of outbreaks, one area for future work would be to investigate if pre-training the model on countries with more cases and fine-tuning it on another country with fewer would produce better results. Model 1 appeared to generate more reliable estimates across a variety of stages of COVID infections, suggesting that generally Model 1 should be used as a default model for all countries first. However, Model 2 seemed to generate better predictions for countries with higher daily cases, suggesting a more parsimonious model could be used instead to achieve better accuracy.

In addition, given the tendency of Model 1 to predict sharp increases when cases were on the decline, any sharp increases predicted by Model 1 should be further substantiated with information on the current situation in the country. As Model 2 tended to overpredict the number of cases during a decline, predictions from model 2 may be taken as an upper bound prediction, rather than the actual number when cases are starting to decline.

As discussed, there are some limitations of this study due to limitations in data availability. Underlying factors may have been missed when data were obtained, adding a degree of uncertainty in the predictions. An example of this is that daily case counts may be drastically high during the forecast phase with the ease of restrictive measures, and these pieces of information may not be present in historical data. It is also unrealistic to fully account for these potential uncertainties, which directly affect the performance of predictive models and cause inaccurate predictions of future cases.

Forecasts can provide potentially useful information to facilitate better allocation of resources and containment planning by healthcare providers and help policymakers manage the consequences of COVID-19 over a longer time horizon. For future work, an ensembling approach to combine both models and potentially other time-series candidate models can be explored. This stems from our observation that a single model might not be able to capture and predict the complex nature of the virus transmission, and a combination of different models will be able to account for the inherent weaknesses of each candidate model. Additionally, for future pandemics involving new variants or viruses, there is potential to apply transfer learning to model them, utilizing the pre-trained bidirectional LSTM developed in this work. This may be able to speed up prediction efforts in a bid to curb the viral spread effectively.

The approach of our study is both model-driven and data driven. While input variables had been selected after literature review on the factors affecting COVID-19 transmission, the data-driven aspect came from the daily data that were provided for each country that was used to fine-tune the model. We believe the data science approach presented in this paper can be generalised for other time-series forecasting applications which use multivariate data.

## Conclusion

A deep-learning approach using Bi-LSTM architecture and open-source data was developed to forecast the new daily number of COVID-19 cases 14 days in advance across 190 countries during the early phase of a pandemic and evaluated using absolute percentage error. The model with fewer variables performed reasonably well compared to the model with more inputs. A deep-learning approach using Bi-LSTM architecture and open-source data can be used as a starting point for forecasting the new daily number of COVID-19 cases 14 days in advance and fewer variables could potentially be used without impacting prediction accuracy.

### Supplementary Information

Supplementary Information.

## Data Availability

The datasets generated during and/or analysed during the current study are available from the Johns Hopkins University research database (github.com/CSSEGISandData/COVID-19), Our World in Data (ourworldindata.com) and OAG (oag.com).

## Code availability

Software application or custom code: The code that support the findings of this study are available from the corresponding author upon reasonable request.
