# Supplementary material for: A novel bidirectional LSTM deep learning approach for COVID-19 forecasting
Source: Sci Rep. 2023 Oct 20;13:17953. doi: 10.1038/s41598-023-44924-8 (PMC10589260; doi:10.1038/s41598-023-44924-8)
Supplement: Supplementary file 1 — Supplementary Information. [file 41598_2023_44924_MOESM1_ESM.pdf]

A novel bidirectional LSTM deep learning approach for COVID-19 forecasting

Nway Nway Aung, MTech [1], Junxiong Pang, Msc, PhD [2], Matthew Chin Heng Chua, BEng, PhD [1], Hui Xing Tan, MTech [1*]

Institute of Systems Science, National University of Singapore [1]

Saw Swee Hock School of Public Health, National University of Singapore [2]

Supplementary Material 1 - All 190 countries and their respective performance with Models 1 and 2

Supplementary Material 2 - Countries which did better under Model 1/Model 2

*Corresponding Author:

Hui Xing Tan (tan.huixing@gmail.com)

NUS Institute of Systems Science

25 Heng Mui Keng Terrace

Singapore 119615

Supplementary Material 1: All 190 countries and their respective performance with Models 1 and 2

			Model 1				Model 2			
S/N	country	Ground Truth	Prediction	MAE	RMSE	Percentage Error	Prediction	MAE	RMSE	Percentage Error
1	Afghanistan	1,691	2,389	47	53	41%	2,521	50	55	49%
2	Albania	15,749	13,294	175	206	16%	13,522	161	201	14%
3	Algeria	5,682	5,074	66	81	11%	4,877	38	44	14%
4	Andorra	1,448	926	33	42	36%	950	33	42	34%
5	Angola	1,730	1,807	39	49	4%	1,479	28	39	15%
6	AntiguaandBarbuda	67	-5	3	5	107%	-6	3	5	109%
7	Argentina	223,887	186,544	2,477	2,937	17%	179,534	2,402	3,031	20%
8	Armenia	5,611	4,430	91	115	21%	10,425	213	245	86%
9	Australia	236	684	24	28	190%	362	15	18	53%
10	Austria	37,605	51,397	802	994	37%	30,227	725	848	20%
11	Azerbaijan	6,802	4,679	453	631	31%	24,850	785	825	265%
12	Bahamas	205	325	10	11	59%	139	8	12	32%
13	Bahrain	8,027	9,617	119	169	20%	7,248	70	93	10%
14	Bangladesh	14,449	20,589	270	316	42%	26,616	529	567	84%

15	Barbados	765	191	30	48	75%	145	32	49	81%
16	Belarus	39,735	42,034	200	305	6%	36,784	277	318	7%
17	Belgium	49,450	48,658	681	792	2%	46,489	610	760	6%
18	Belize	706	1,598	39	43	126%	1,729	44	48	145%
19	Benin	589	5	26	52	99%	3	26	52	99%
20	Bhutan	88	135	6	9	53%	86	4	8	2%
21	Bolivia	45,681	23,842	978	1,170	48%	22,848	995	1,146	50%
22	BosniaandHerzegovina	6,577	3,342	272	385	49%	9,879	245	303	50%
23	Botswana	5,243	855	245	446	84%	373	236	455	93%
24	Brazil	1,191,023	924,402	18,811	19,659	22%	927,888	18,078	18,908	22%
25	Brunei	7	1	0	1	82%	-2	0	1	126%
26	Bulgaria	10,736	19,015	452	634	77%	19,466	410	516	81%
27	BurkinaFaso	2,816	3,231	57	73	15%	3,247	80	99	15%
28	Burma	10,662	13,404	150	202	26%	13,641	133	168	28%
29	Burundi	747	69	30	38	91%	64	30	39	91%
30	CaboVerde	1,833	1,011	36	41	45%	756	48	53	59%

31	Cambodia	79	26	3	5	68%	23	3	5	71%
32	Cameroon	2,769	-15	123	377	101%	606	140	370	78%
33	Canada	133,537	170,835	1,756	2,055	28%	183,175	2,168	2,538	37%
34	CentralAfricanRepublic	12	120	5	5	897%	4	2	3	63%
35	Chad	957	386	25	31	60%	299	29	34	69%
36	Chile	93,728	60,041	1,465	1,526	36%	70,391	1,015	1,120	25%
37	China	3,501	1,700	78	91	51%	1,704	79	92	51%
38	Colombia	339,316	272,388	3,587	4,016	20%	277,990	3,249	3,907	18%
39	Comoros	1,676	12	72	98	99%	61	71	96	96%
40	CongoBrazzaville	760	165	38	116	78%	98	36	117	87%
41	CongoKinshasa	3,802	3,882	67	97	2%	2,653	68	102	30%
42	CostaRica	15,662	26,983	669	808	72%	28,728	719	851	83%
43	CotedIvoire	5,145	1,497	164	192	71%	1,160	177	204	77%
44	Croatia	14,480	5,260	426	528	64%	33,766	839	920	133%
45	Cuba	12,863	7,126	261	304	45%	6,495	283	360	50%
46	Cyprus	4,202	13,191	391	421	214%	10,881	291	315	159%

47	Czechia	175,173	274,800	4,782	5,854	57%	153,931	2,441	2,753	12%
48	Denmark	19,986	40,165	905	990	101%	46,316	1,156	1,233	132%
49	Djibouti	67	-2	3	4	103%	-1	3	4	102%
50	Dominica	11	-1	1	1	109%	-1	1	1	108%
51	DominicanRepublic	35,522	19,335	704	777	46%	18,615	735	812	48%
52	Ecuador	31,680	19,851	730	1,068	37%	21,343	709	1,051	33%
53	Egypt	18,141	28,787	463	494	59%	26,395	359	397	45%
54	ElSalvador	6,711	2,106	290	382	69%	3,739	277	342	44%
55	EquatorialGuinea	227	-79	13	21	135%	-143	16	23	163%
56	Eritrea	579	142	26	52	75%	42	25	54	93%
57	Estonia	11,671	11,941	147	163	2%	11,411	152	175	2%
58	Eswatini	4,736	5,104	77	89	8%	3,602	93	109	24%
59	Ethiopia	10,078	8,203	118	152	19%	9,177	113	153	9%
60	Fiji	2	0	0	0	92%	-1	0	0	139%
61	Finland	7,170	4,780	113	144	33%	6,144	97	114	14%
62	France	451,177	312,163	11,397	14,419	31%	276,680	12,842	14,312	39%

63	Gabon	1,054	264	47	72	75%	264	48	72	75%
64	Gambia	252	17	11	18	93%	33	10	18	87%
65	Georgia	22,083	38,530	759	952	74%	44,744	1,021	1,205	103%
66	Germany	320,021	227,119	6,779	8,649	29%	315,722	6,583	8,395	1%
67	Ghana	11,549	1,106	493	684	90%	1,918	470	648	83%
68	Greece	13,463	16,378	221	291	22%	17,724	224	297	32%
69	Grenada	21	7	1	2	67%	-3	1	3	114%
70	Guatemala	17,440	13,261	398	458	24%	13,634	382	441	22%
71	Guinea	642	551	17	22	14%	597	18	21	7%
72	GuineaBissau	179	-36	9	24	120%	-24	9	24	113%
73	Guyana	1,172	425	33	39	64%	464	32	37	60%
74	Haiti	1,330	157	56	75	88%	59	56	76	96%
75	Honduras	22,286	7,874	627	660	65%	7,281	652	688	67%
76	Hungary	29,843	35,489	558	746	19%	43,916	694	871	47%
77	Iceland	135	307	9	11	127%	391	12	15	190%
78	India	344,193	334,051	3,728	5,324	3%	295,560	3,453	5,578	14%

79	Indonesia	269,974	187,557	3,583	3,921	31%	182,837	3,789	4,146	32%
80	Iran	143,485	138,544	533	650	3%	155,993	606	737	9%
81	Iraq	18,212	15,272	193	225	16%	11,969	289	346	34%
82	Ireland	60,663	15,082	2,018	2,524	75%	6,378	2,360	2,716	89%
83	Israel	166,078	194,961	2,544	3,117	17%	120,102	2,618	3,428	28%
84	Italy	315,142	347,217	3,566	4,100	10%	349,663	2,893	3,616	11%
85	Jamaica	2,242	1,400	42	53	38%	1,631	36	46	27%
86	Japan	115,219	88,893	1,538	1,877	23%	101,728	1,591	1,812	12%
87	Jordan	21,692	31,580	455	501	46%	43,742	959	997	102%
88	Kazakhstan	28,161	19,038	415	505	32%	23,290	301	369	17%
89	Kenya	2,819	8,046	227	261	185%	7,186	190	204	155%
90	KoreaSouth	10,509	11,353	160	200	8%	14,003	218	260	33%
91	Kosovo	6,954	3,305	280	368	52%	5,416	221	286	22%
92	Kuwait	11,784	7,411	195	212	37%	9,782	97	121	17%
93	Kyrgyzstan	2,597	658	108	134	75%	- 658	144	163	125%
94	Laos	3	2	0	1	24%	2	0	1	46%

95	Latvia	18,459	20,573	285	368	11%	17,689	230	280	4%
96	Lebanon	90,913	72,354	1,231	1,639	20%	87,730	896	1,136	4%
97	Lesotho	3,494	102	150	262	97%	195	149	260	94%
98	Liberia	160	- 12	7	21	108%	49	7	20	69%
99	Libya	14,629	11,640	314	373	20%	11,859	299	351	19%
100	Liechtenstein	182	467	13	14	157%	443	11	12	143%
101	Lithuania	26,000	46,601	1,022	1,097	79%	47,529	1,082	1,160	83%
102	Luxembourg	2,644	4,276	126	147	62%	4,865	119	145	84%
103	Madagascar	1,298	- 35	61	136	103%	- 267	68	145	121%
104	Malawi	16,038	598	674	744	96%	751	667	738	95%
105	Malaysia	83,851	49,767	1,566	1,890	41%	48,730	1,527	1,817	42%
106	Maldives	1,801	792	45	55	56%	730	47	58	59%
107	Mali	579	1,319	33	37	128%	915	20	22	58%
108	Malta	3,936	3,152	42	49	20%	2,942	46	57	25%
109	MarshallIslands	-	- 0	0	0	NA	0	0	0	NA
110	Mauritania	1,283	1,542	42	48	20%	2,458	52	59	92%

111	Mauritius	30	11	2	3	65%	8	1	3	75%
112	Mexico	356,329	240,925	5,884	6,907	32%	240,771	5,657	6,637	32%
113	Micronesia	1	-0	0	0	106%	-0	0	0	113%
114	Moldova	11,135	13,038	223	266	17%	18,292	311	369	64%
115	Monaco	448	429	12	15	4%	308	10	13	31%
116	Mongolia	406	324	7	10	20%	272	8	10	33%
117	Montenegro	10,063	10,039	56	68	0%	10,692	66	78	6%
118	Morocco	20,936	37,287	711	857	78%	34,807	603	677	66%
119	Mozambique	18,172	2,243	693	729	88%	2,570	678	715	86%
120	MSZaandam	-	-1	0	0	NA	-1	0	0	NA
121	Namibia	6,689	8,107	111	136	21%	5,846	85	110	13%
122	Nepal	6,800	-812	351	398	112%	4,430	172	223	35%
123	Netherlands	121,012	161,613	1,773	2,067	34%	176,486	2,596	2,884	46%
124	NewZealand	116	16	5	8	86%	42	5	8	64%
125	Nicaragua	156	-5	7	19	103%	-37	8	19	124%
126	Niger	770	1,116	23	27	45%	1,212	24	32	57%

127	Nigeria	33,764	24,291	561	728	28%	13,225	893	984	61%
128	NorthMacedonia	7,099	10,355	158	211	46%	10,632	169	210	50%
129	Norway	8,490	12,295	199	235	45%	12,205	185	210	44%
130	Oman	4,256	4,053	155	212	5%	4,601	144	184	8%
131	Pakistan	46,911	54,106	501	582	15%	46,709	437	539	0%
132	Panama	47,342	76,302	1,421	1,683	61%	67,771	1,048	1,150	43%
133	PapuaNewGuinea	40	-8	2	4	120%	-5	2	4	111%
134	Paraguay	19,233	19,666	221	267	2%	20,481	161	187	6%
135	Peru	112,059	39,658	4,718	6,245	65%	1,815	5,026	7,118	98%
136	Philippines	41,766	27,844	605	686	33%	21,242	892	953	49%
137	Poland	147,740	221,857	3,531	4,644	50%	245,252	4,240	4,807	66%
138	Portugal	253,807	103,978	6,514	7,041	59%	127,786	5,479	6,288	50%
139	Qatar	5,869	12,359	286	312	111%	5,501	51	63	6%
140	Romania	64,944	99,682	1,512	1,670	53%	101,520	1,602	1,769	56%
141	Russia	487,185	512,598	2,285	2,749	5%	528,872	2,483	2,967	9%
142	Rwanda	5,936	2,995	130	161	50%	2,630	147	172	56%

143	SaintKittsandNevis	3	-1	0	0	133%	1	0	0	60%
144	SaintLucia	800	44	33	47	94%	67	34	46	92%
145	SaintVincentandtheGrenadines	752	3	33	49	100%	-2	33	49	100%
146	SanMarino	397	805	19	22	103%	890	24	34	124%
147	SaoTomeandPrincipe	203	2	9	11	99%	14	8	11	93%
148	SaudiArabia	4,492	584	170	189	87%	1,625	128	142	64%
149	Senegal	5,851	3,150	118	135	46%	3,715	99	112	37%
150	Serbia	39,138	58,724	1,063	1,442	50%	68,412	1,346	1,482	75%
151	Seychelles	754	28	32	52	96%	18	32	53	98%
152	SierraLeone	811	220	27	41	73%	158	29	43	81%
153	Singapore	700	699	17	22	0%	996	17	21	42%
154	Slovakia	48,749	49,781	709	897	2%	47,859	709	867	2%
155	Slovenia	29,844	40,091	571	748	34%	45,153	752	927	51%
156	SolomonIslands	-	-0	0	0	NA	1	0	0	NA
157	Somalia	58	-3	3	8	106%	-17	4	8	129%
158	SouthAfrica	261,191	300,036	4,753	5,618	15%	247,097	3,904	5,098	5%

159	SouthSudan	340	50	15	34	85%	1	15	35	100%
160	Spain	692,759	256,826	26,974	33,565	63%	205,444	28,834	35,293	70%
161	SriLanka	16,852	12,418	199	227	26%	13,400	157	197	20%
162	Sudan	3,101	3,742	157	256	21%	3,566	153	264	15%
163	Suriname	1,584	1,010	26	32	36%	888	30	36	44%
164	Sweden	77,486	40,482	3,216	4,361	48%	23,315	3,278	4,853	70%
165	Switzerland	43,337	79,678	1,894	2,196	84%	125,957	3,740	4,148	191%
166	Syria	1,869	2,224	18	23	19%	2,438	25	31	30%
167	Taiwan	86	70	2	3	19%	66	2	3	24%
168	Tajikistan	3	511	22	22	16928%	502	22	22	16633%
169	Tanzania	-	23	1	1	NA	29	1	1	NA
170	Thailand	9,565	1,216	376	550	87%	1,241	376	550	87%
171	TimorLeste	21	-1	1	2	103%	0	1	2	99%
172	Togo	1,180	574	32	47	51%	770	30	45	35%
173	TrinidadandTobago	345	375	6	7	9%	-562	39	42	263%
174	Tunisia	53,982	50,503	657	865	6%	44,824	656	870	17%

175	Turkey	169,882	452,440	12,285	12,434	166%	147,561	1,687	2,007	13%
176	Uganda	2,283	8,792	289	302	285%	6,576	203	224	188%
177	Ukraine	121,746	143,264	1,580	1,851	18%	156,017	1,975	2,231	28%
178	UnitedArabEmirates	78,905	32,988	1,996	2,021	58%	36,719	1,834	1,871	53%
179	UnitedKingdom	861,939	1,373,007	27,386	32,037	59%	836,075	13,602	17,024	3%
180	UnitedStates	4,224,535	5,028,886	44,250	52,940	19%	5,011,509	47,506	55,796	19%
181	Uruguay	17,399	22,202	317	354	28%	13,521	215	308	22%
182	Uzbekistan	1,226	-797	90	99	165%	-179	61	70	115%
183	Vanuatu	-	0	0	0	NA	0	0	0	NA
184	Venezuela	11,260	4,645	288	301	59%	5,366	256	273	52%
185	Vietnam	305	101	13	31	67%	91	13	31	70%
186	WestBankandGaza	12,888	22,651	424	463	76%	23,776	473	490	84%
187	WesternSahara	-	0	0	0	NA	0	0	0	NA
188	Yemen	17	-2	1	1	115%	-13	1	2	175%
189	Zambia	28,891	6,258	984	1,030	78%	4,128	1,077	1,120	86%
190	Zimbabwe	13,728	17,814	650	754	30%	6,953	469	556	49%

Supplementary Material 2: Countries which did better under Model 1/Model 2

Better performance using Model 1 – Moldova, Jordan, Croatia, Switzerland

Moldova

Model 1	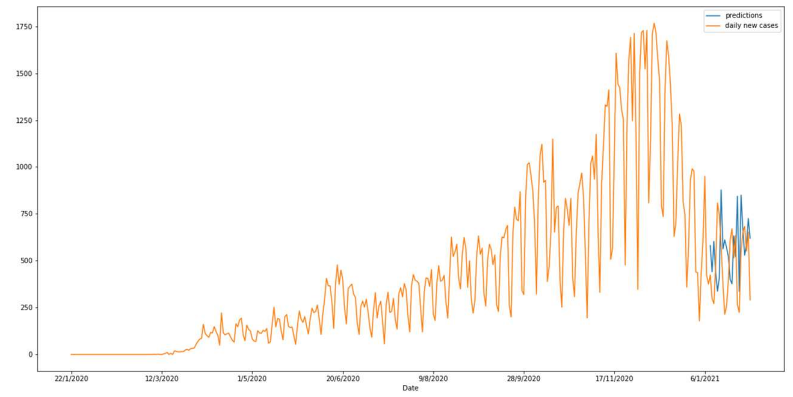
Model 2	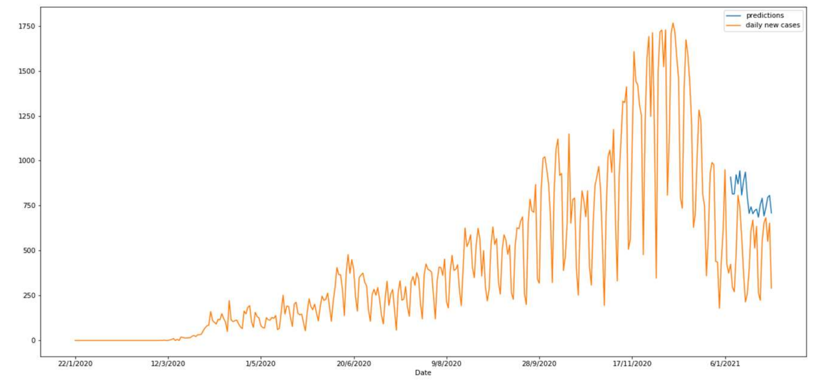

Jordan

Model 1	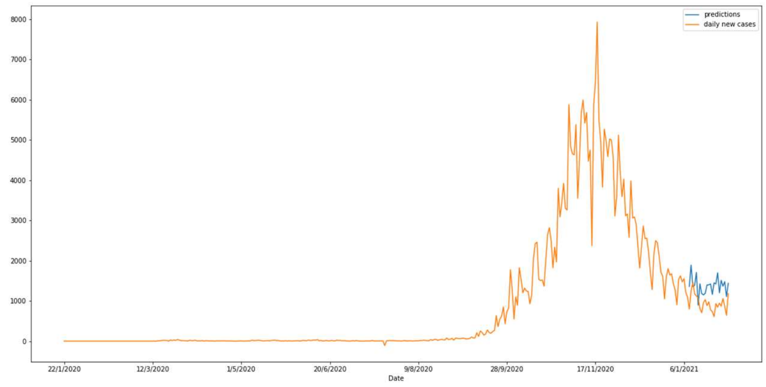
Model 2	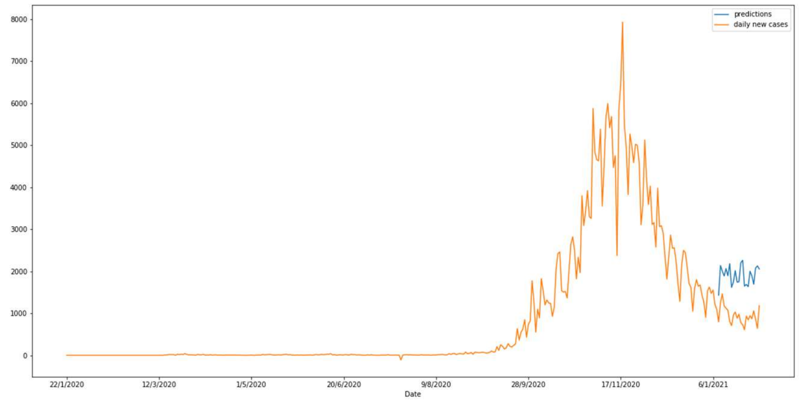

Croatia

Model 1	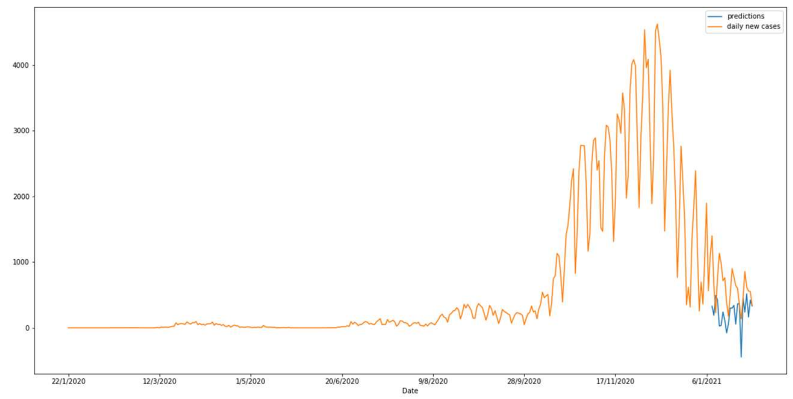
Model 2	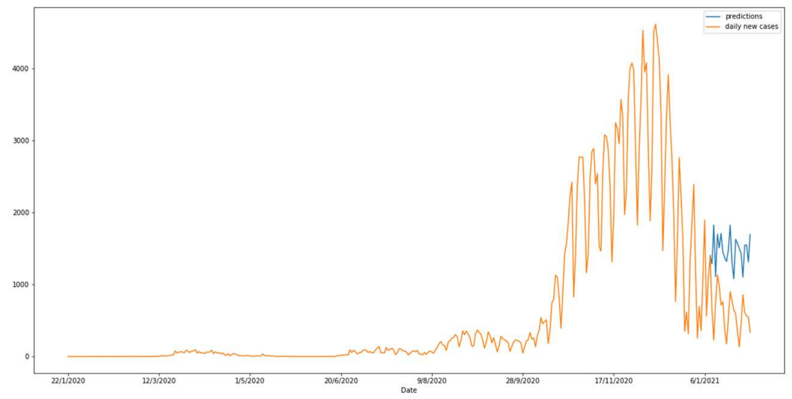

Switzerland

<table>
<tr><td>Model 1</td><td>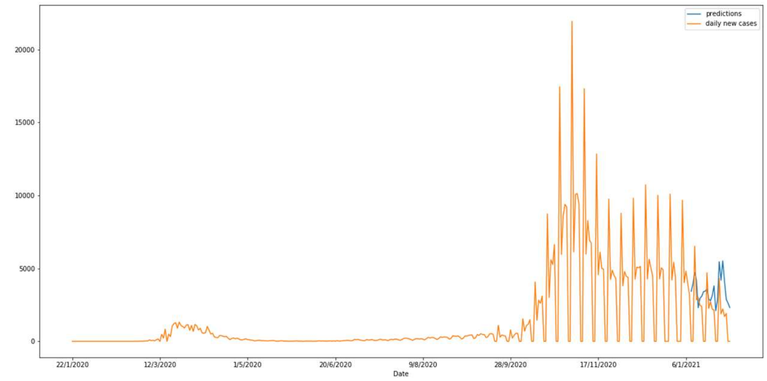
</td></tr>
<tr><td>Model 2</td><td>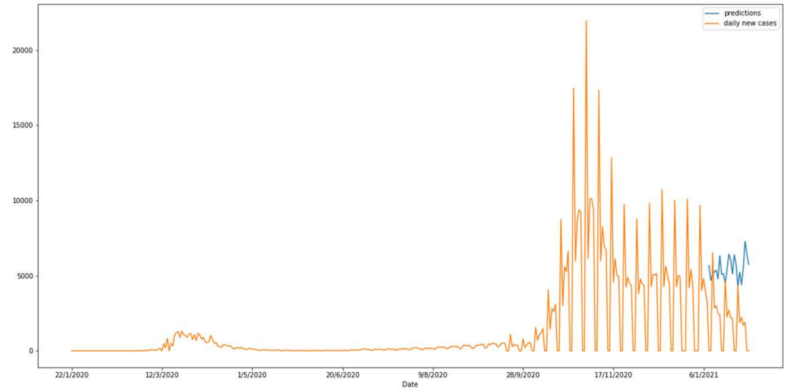
</td></tr>
</table>

Better performance using Model 2 – Czechia, UK, Turkey, Germany

Czechia

<table>
<tr>
<td>Model 1</td>
<td>
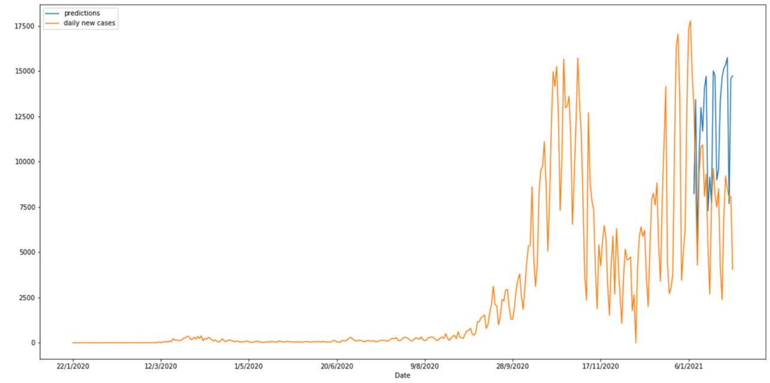

</td>
</tr>
<tr>
<td>Model 2</td>
<td>
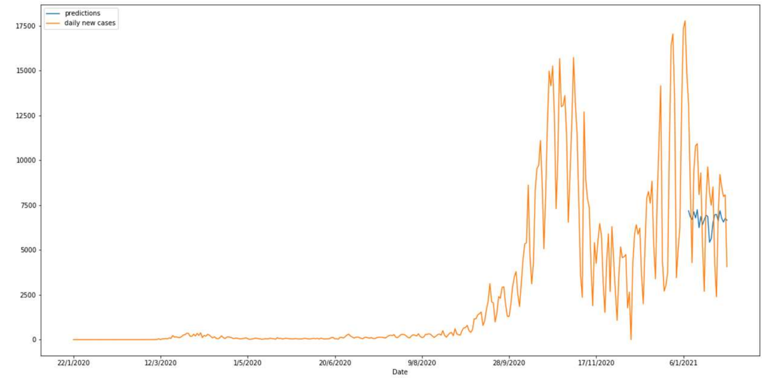

</td>
</tr>
</table>

UK

<table>
<tr><td>Model 1</td><td>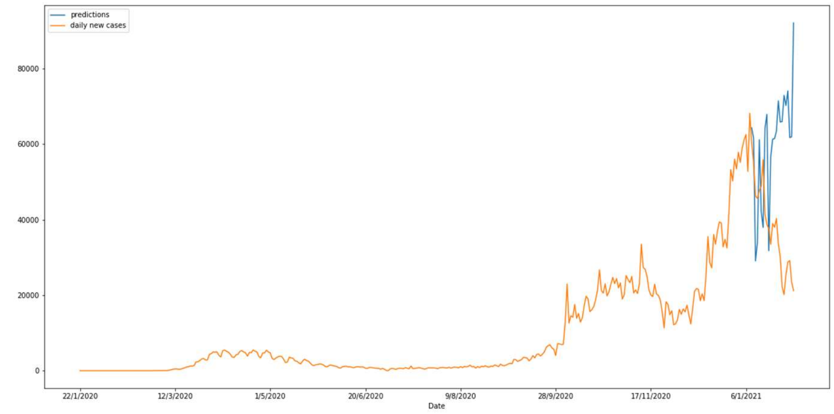
</td></tr>
<tr><td>Model 2</td><td>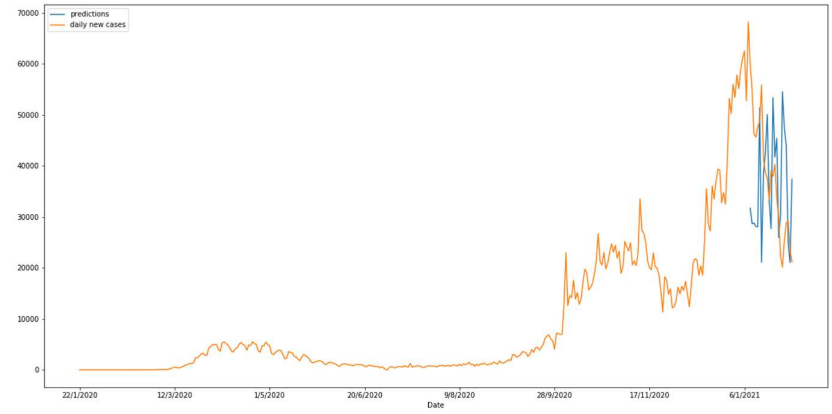
</td></tr>
</table>

Turkey

Model 1	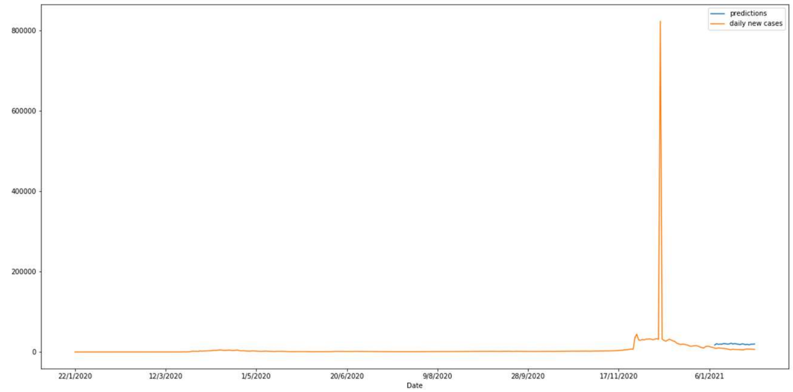
Model 2	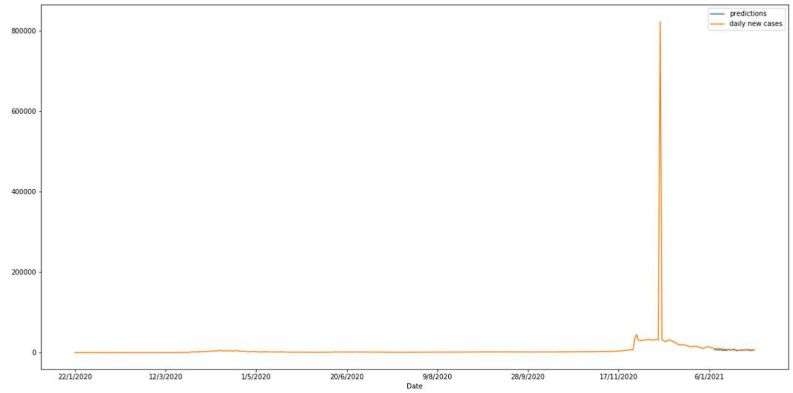

Germany

Model 1	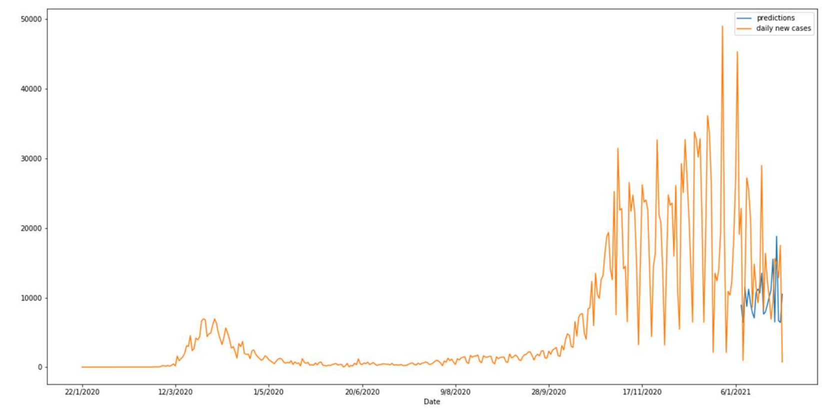
Model 2	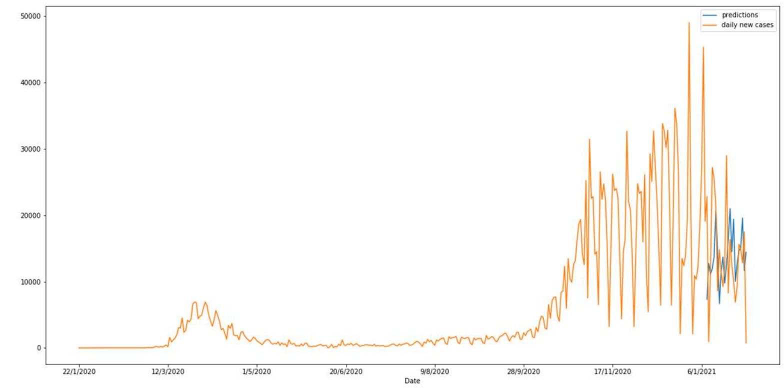
